# Periocular cutaneous anthrax in Jimma Zone, Southwest Ethiopia: a case series

**DOI:** 10.1186/1756-0500-6-313

**Published:** 2013-08-07

**Authors:** Yeshigeta Gelaw, Tsedeke Asaminew

**Affiliations:** 1 Department of Ophthalmology, College of Public Health and Medical Sciences, Jimma University, PO Box: 440, Jimma, Ethiopia

**Keywords:** Anthrax, Preseptal cellulitis, *Bacillus anthracis*, Zoonotic disease, Eye lid

## Abstract

**Background:**

Anthrax is a zoonotic disease caused by *Bacillus anthracis*. Naturally occurring human infection is rare and is generally the result of contact with anthrax-infected animals or animal products.

**Case presentation:**

We examined three patients who had contact with presumed anthrax-infected animal and/or its product and presented with preseptal cellulitis with a localized itchy erythematous papule of the eyelid and non-pitting periorbital edema, followed by ulceration and dark eschar formation. All the three patients responded to intravenous antibiotics, and the lesion resolved leaving scars which caused cicatricial ectropion in all cases.

**Conclusion:**

Anthrax is a rare disease but should be considered in the differential diagnosis of ulcerative (and eschar forming) preseptal cellulitis with a history of contact with anthrax-infected animals or animal products. Furthermore, cicatrization of the eyelids, one of the sequelae of periocular cutaneous anthrax, should be addressed. Urgent case report to the local zoonotic disease and infection control body and other responsible authorities is recommended.

## Background

Anthrax is caused by *Bacillus anthracis*, a gram-positive, nonmotile, spore-forming rod that is found in soil and predominantly causes disease in herbivores such as cattle, goats, and sheep. Anthrax spores can remain viable for decades and this remarkable stability of the spores makes them an ideal bioweapon [1]. Anthrax is transmitted via direct or indirect contact with infected animals and their products, such as hides or wool, and inhalation of spores [1]. Since farmers, butchers, veterinarians, shepherds, and farm workers are at great risk of exposure to infected material, anthrax is also considered as an occupational disease often limited to underdeveloped countries [2]. It is, therefore, a common disease in parts of Africa, Latin America, Asia and Eastern Europe [3].

The three major clinical forms of anthrax are gastrointestinal, cutaneous, and inhalational. Cutaneous anthrax is the most common form accounting for about 95% of the cases and it mostly occurs in exposed skin areas, like the face, neck and forearms. In the face, the eyelid is the most common site of infection [4-6].

Before the advent of antibiotics, and today in rural areas of the developing world where antibiotics are often not available, cutaneous anthrax may lead to septicemia, with a mortality rate of 20–30%. If treated appropriately and timely though, mortality is less than 1%. Eyelid anthrax, in particular, can lead to serious complications like cicatricial ectropion resulting in corneal scar and blindness in the affected eye [7-9].

To our knowledge there are no recent case reports or researches done on cutaneous anthrax in Ethiopia and/or the region. This paper, thus, reports a case series of 3 patients with periocular anthrax that were seen at Jimma University Specialized Hospital, Ethiopia from June 2011 to May 2012, and will highlight the presence of infectious zoonotic disease of public health importance for coordinated preventive and therapeutic measures in developing countries.

## Case presentation

### Case 1

A 29-year-old male farmer from Sigmo District, Jimma Zone, Ethiopia presented to the Eye Clinic with a complaint of right side severe periocular swelling of 10 days duration. The swelling started as a small reddish raised lesion on his right upper eye lid which was later followed by vesicle formation and dark ulcerative wound with progressive swelling involving the upper lid, lower lid and cheek on the same side. He slaughtered an ill ox in his village a week before the start of his illness and he also ate the uncooked meat. There was no history of trauma or surgery. He was given oral antibiotics from the local pharmacy which he took for a week prior to presentation to the Eye Clinic.

**Figure 1:**
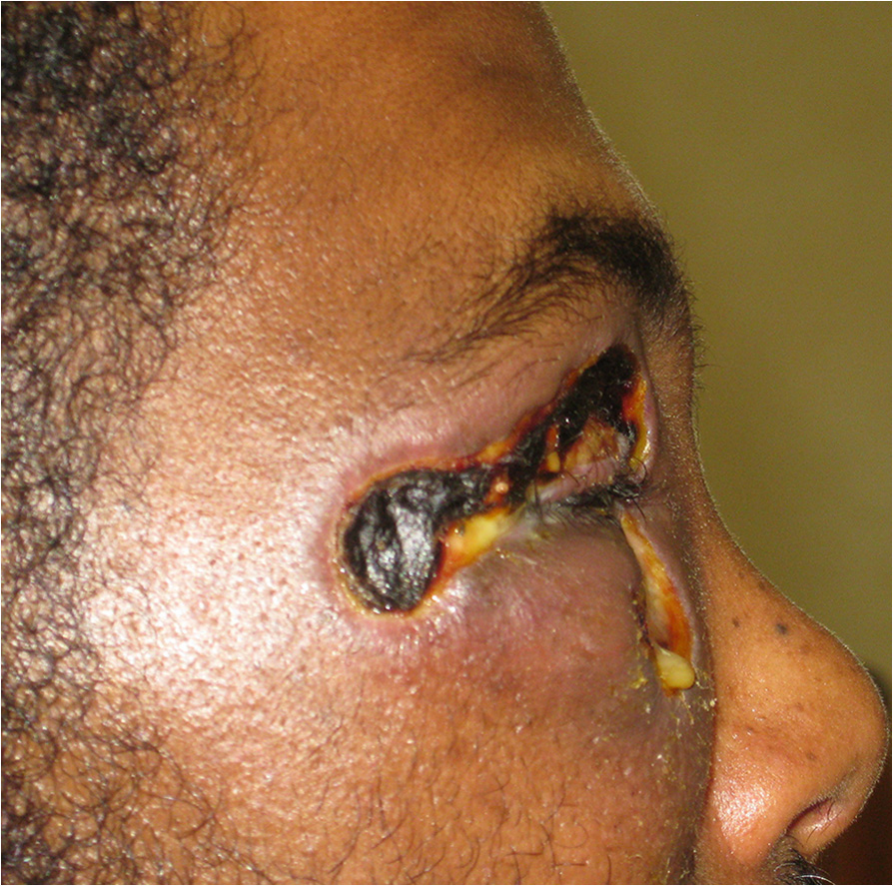
Eschar forming lesion.

On examination, the patient had diffuse non-pitting edema of both the upper lid and lower lid with ulcerated central dark tissue. It was not tender to touch and there was no abscess (Figure 1). The patient was unable to open the affected lids and it was thus difficult to record visual acuity at presentation but the cornea looked normal. Vital signs were normal and his body temperature was 36.1°C; and the white blood cell (WBC) count was 7, 800 × 10^3^.

**Figure 2:**
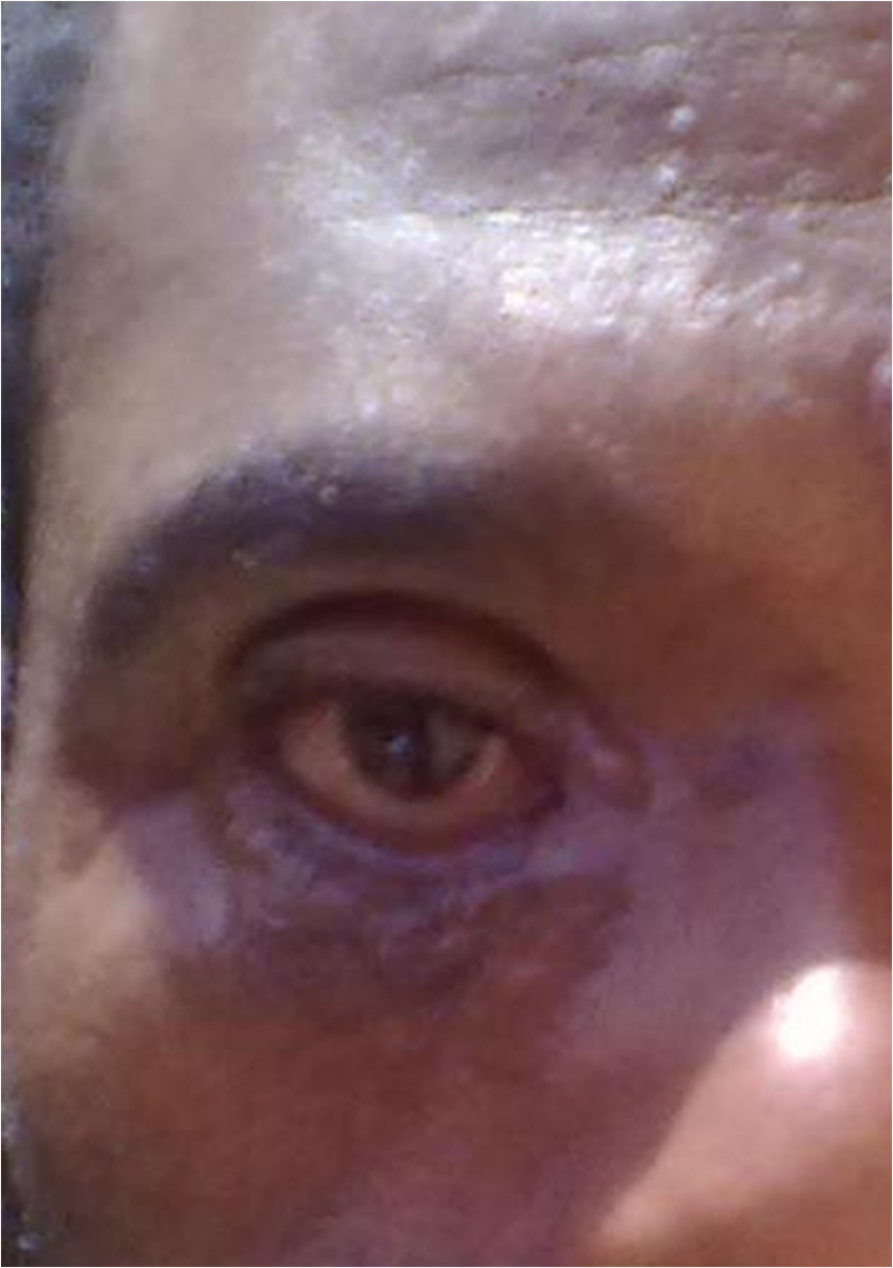
Cicatricial ectropion.

With the clinical diagnosis of severe preseptal cellulitis/periocular cutaneous anthrax/, the patient was admitted to the Eye Ward and started on intravenous ceftriaxone 1gm BID and cloxacilline 500 mg IV QID for 2 weeks and then he was put on oral doxycycline 100 mg BID for 6 weeks. After 3 weeks the lesion resolved and visual acuity was 6/6. There was persistent cicatricial ectropion of the right eyelids (Figure 2) for which full thickness skin graft was done at 2 months of follow up. On further follow up at one year, there was lid disfigurement on the nasal part of both lids which warranted surgical correction.

### Case 2

A 45-year-old male farmer patient from Sigmo District, Jimma Zone, Ethiopia started to have a small ulcer over the right eye brow which became progressively swollen to involve the upper eye lid, lower eye lid and face on the same side within 5 days. He slaughtered an ox and ate uncooked beef meat. He also knew a person who had similar illness in the same vicinity. There was no history of trauma or surgery.

On examination, he had diffuse marked edema on the upper, lower eye lid and face on the same side. There was also dark colored ulcer on the upper and lower eye lid, and this made initial visual acuity measurement and further examination of intraocular structures difficult. There was no abscess and the edema was non-pitting and non-tender. He took oral antibiotics for 3 days and his body temperature was normal.

The patient was admitted with the impression of preseptal cellulitis to rule out orbital cellulitis and was started on intravenous ceftriaxone 1 gm BID and cloxacilline 500 mg QID. After a day the clinical diagnosis was changed to cutaneous anthrax as the skin lesion became more distinguishable and typical with black eschar formation. Thus, oral doxycycline 100 mg BID was added and continued for 8 weeks. After 2 weeks of illness, the swelling decreased and the skin of both the lower and upper lids became necrotic. The eye exam was normal and visual acuity was 6/6. After 2 months he developed lower lid ectropion for which surgical correction was done at 10 weeks of follow up.

### Case 3

**Figure 3:**
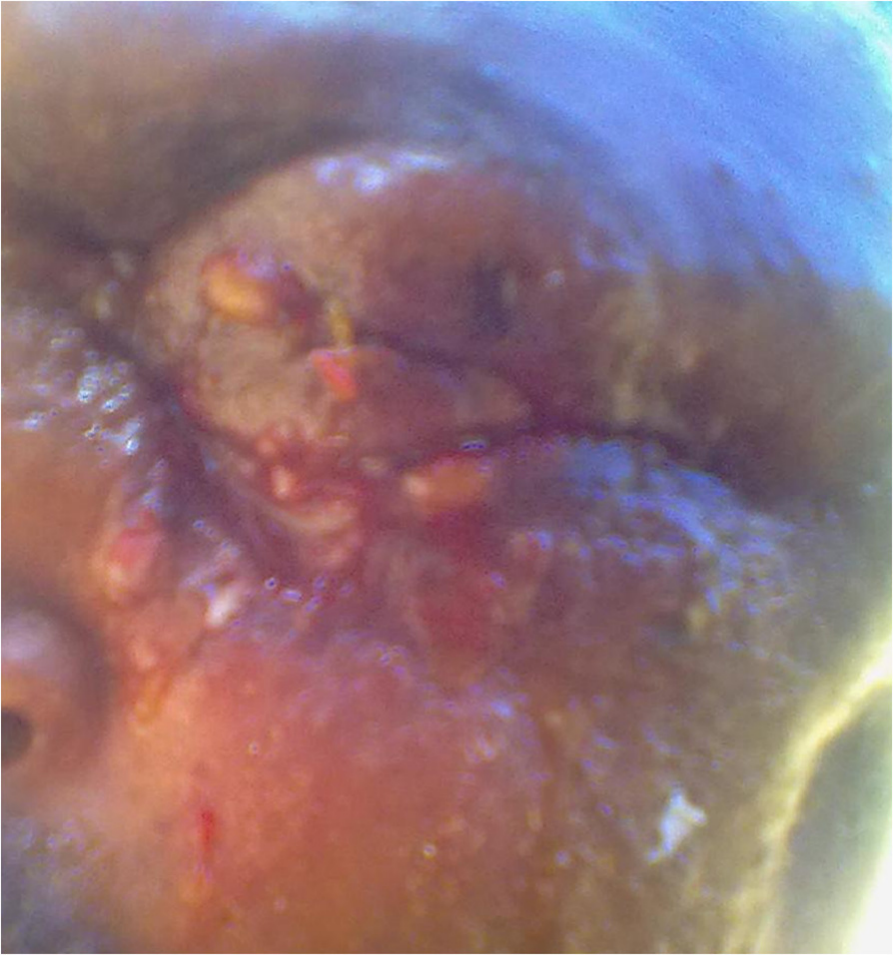
Diffuse periorbital edema.

A 25-year-old male patient from Agaro District, Jimma Zone, Ethiopia started to have small itchy red skin lesion below the left eye lid. Within 5 days the swelling progressed to involve the upper lid, cheek and lips on the left side associated with dark ulceration over the swelling on both eyelids as shown in Figure 3. There was no history of trauma or surgery but there was a history of direct contact with sick ox while giving medicine 1 week prior to the onset of his illness.

**Figure 4:**
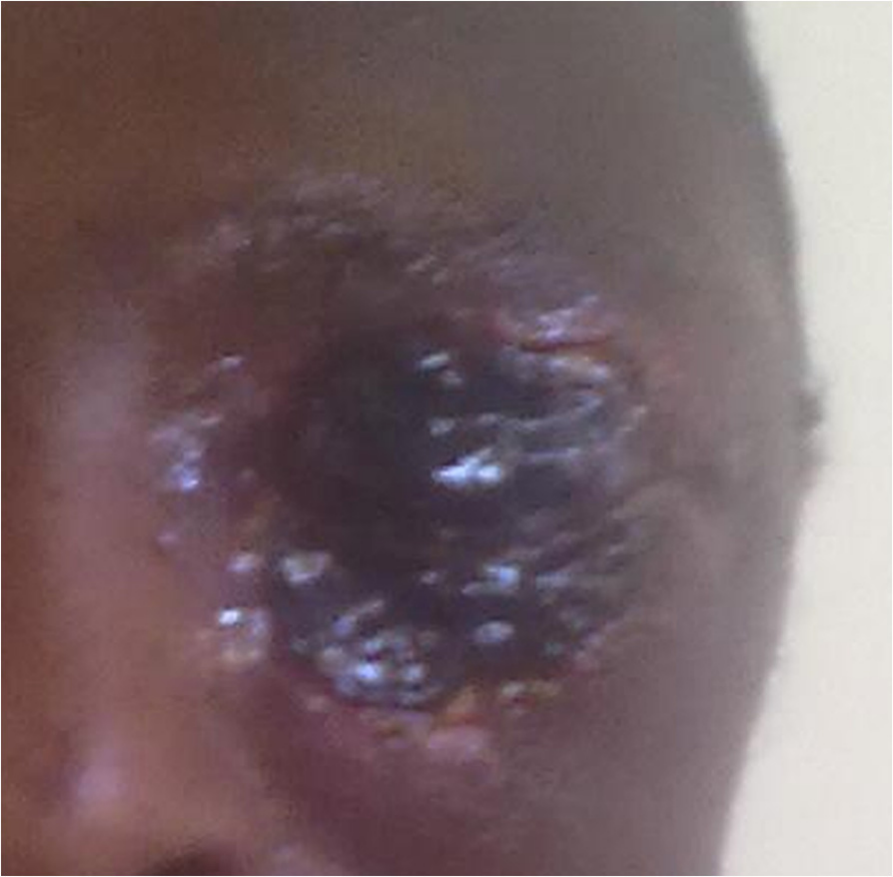
Black eschar with reduction of periorbital edema.

On examination, he had diffuse edema on the left upper lid, lower lid and left part of the face with dark ulcer on the upper and lower lids. Visual acuity and other ocular examination were difficult initially. The temperature was 38.8°C. He took Augmentin 625 mg PO TID for 24 hours prior to presentation. The patient was admitted and started on intravenous ceftriaxone 1 gm BID and cloxacilline 500 mg QID. After 2 days there was mild decrement of the edema and the skin lesion became more discernable, thus doxycycline 100 mg PO BID was given. Black eschar began to form subsequently with resolution of the edema as seen in Figure 4. After 1 month of treatment, there was inter-palpebral height shortening with scarring resulting in difficulty of opening the eye lids. Mild ectropion on the lower eye lid was also present. The patient will be followed until the scar is stabilized enough to do skin graft.

### Discussion

Although preseptal cellulitis due to anthrax is a rare disease, it should be considered in the differential diagnosis of a patient who has close contact with animals [7,8]. Most reported anthrax cases with eyelid involvement have close contact history with animal or animal products [5,7-10] and at the initial stage of this disease such a typical history is very helpful in the diagnosis. In severe cases of preseptal cellulitis where there is diffuse swelling and inflammation it might be difficult to discern a typical skin lesion for cutaneous anthrax. Nevertheless, patients may give history of a skin lesion with characteristic evolution [10].

The cutaneous anthrax lesion commences as a minute red macule which then becomes a tiny papule or pimple, heralded by pruritis, often present before evidence of any visible lesion. These papules then enlarge to take on a pinkish fleshy appearance followed by formation of fine, tight, shiny yellow or pink vesicles. Then a non-pitting brawny edema begins to appear; and satellite vesicles or blisters may also appear. By about 48 hours the central vesicle ruptures forming a moist ulcer, with a reddish or brownish base and later the base of the central ulcer darkens and by the fourth day, it is black and is surrounded by a raised erythematous indurated ring of tissue. At this stage the lesion is characteristic of anthrax [10]. As the central eschar becomes tougher and drier, it assumes a more carbon-like appearance [10]. This evolution of skin lesion was in agreement with the clinical description in our patients and hence supports the diagnosis of periocular cutaneous anthrax.

It is, therefore, vital to have high index of suspicion especially in those cases accompanied by history of close contact with animals or animal products. Though the majority of diagnoses could probably be made on clinical grounds alone [11], gram staining and culture can confirm the microbiological diagnosis of anthrax as a cause of preseptal cellulitis. However, isolation of *B.anthracis* was not possible in our cases as all presented late to our Eye Clinic and had already been on oral antibiotics for 2–7 days which could eradicate *B.anthracis* from the ulcerative wound/necrotic tissue. Cultures taken from the lesions of patients treated with penicillin become negative within a few hours after treatment initiation [12] and the diagnosis of cutaneous anthrax, therefore, relies mainly on clinical grounds.

The other possible differential diagnosis for periocular cutaneous anthrax is necrotizing fasciitis which presents with rapidly progressive severe inflammation initially involving subcutaneous tissues and later the skin and accompanied by tissue necrosis [13,14]. It classically involves the trunk, groin/perineum, and lower limbs, as well as postoperative wound sites. However, primary involvement of the eyelids is also possible and usually secondary to trauma, surgery or immunosuppression [13-15]. Periocular necrotizing fasciitis is characterized by periorbital redness and edema leading to formation of large bullae and typical changes in the skin color progressing from rose coloration to blue-gray owing to gangrene from underlying thrombosis [14,15]. Disproportionate complaints of pain and anesthesia over the affected area are other suggestive features of necrotizing fasciitis [15] which were not found in our cases. The swelling around the lesion is also exquisitely tender [13] unlike the finding in our cases. Black eschar is also not characteristic of necrotizing fasciitis [13] but was seen in all of our cases.

Cicatricial ectropion is one of the most serious complications of periocular anthrax [7-10]. Yorston and Foster reported that 8 of 11 patients with cutaneous anthrax of the eyelid had developed cicatricial ectropion despite appropriate therapy including high doses of penicillin. The ratio of the long-term sequelae of the disease is high despite antimicrobial therapy [7]. Ectropion correction needs to be done after complete cure of the wound mostly after 6 months [7-10]. Our patients also developed ectropion despite antimicrobial therapy. For our patients, correction of ectropion was done earlier than mentioned in the literature and none of them had corneal ulceration or scar and cosmetic outcomes were acceptable.

## Conclusions

When preseptal cellulitis is encountered in a patient who is in close contact with animals and/or history of characteristic skin lesion, cutaneous eyelid anthrax should always be considered in the differential diagnosis and appropriate treatment be initiated promptly. Cicatricial ectropion being a common sequela of cutaneous eyelid anthrax should also be addressed. Anthrax being a zoonotic disease, case report should be made to responsible bodies for surveillance and control measures.

## Consent

Written informed consents were obtained from the patients for publication of this Case series and any accompanying images. Copies of the written consents are available for review by the Editor-in-Chief of this journal.

## Competing interests

The authors declare that they have no competing interests.

## Authors’ contributions

YG collected and interpreted the data and wrote the manuscript. TA collected and interpreted the data and assisted draft the manuscript. Both authors read and approved the final manuscript.
